# Mutants in *Drosophila* TRPC Channels Reduce Olfactory Sensitivity to Carbon Dioxide

**DOI:** 10.1371/journal.pone.0049848

**Published:** 2012-11-19

**Authors:** Farhath Badsha, Pinky Kain, Sunil Prabhakar, Susinder Sundaram, Raghu Padinjat, Veronica Rodrigues, Gaiti Hasan

**Affiliations:** 1 National Centre for Biological Sciences, Tata Institute of Fundamental Research, Bangalore, Karnataka, India; 2 Inositide Laboratory, Babraham Institute, Cambridge, United Kingdom; 3 Department of Biological Sciences, Tata Institute of Fundamental Research, Mumbai, Maharashtra, India; Monell Chemical Senses Center, United States of America

## Abstract

**Background:**

Members of the canonical Transient Receptor Potential (TRPC) class of cationic channels function downstream of Gαq and PLCβ in *Drosophila* photoreceptors for transducing visual stimuli. Gαq has recently been implicated in olfactory sensing of carbon dioxide (CO_2_) and other odorants. Here we investigated the role of PLCβ and TRPC channels for sensing CO_2_ in *Drosophila*.

**Methodology/Principal Findings:**

Through behavioral assays it was demonstrated that *Drosophila* mutants for *plc21c*, *trp* and *trpl* have a reduced sensitivity for CO_2_. Immuno-histochemical staining for TRP, TRPL and TRPγ indicates that all three channels are expressed in *Drosophila* antennae including the sensory neurons that express CO_2_ receptors. Electrophysiological recordings obtained from the antennae of protein null alleles of TRP (*trp^343^)* and TRPL (*trpl^302^)*, showed that the sensory response to multiple concentrations of CO_2_ was reduced. However, *trpl^302^; trp^343^* double mutants still have a residual response to CO_2_. Down-regulation of TRPC channels specifically in CO_2_ sensing olfactory neurons reduced the response to CO_2_ and this reduction was obtained even upon down-regulation of the TRPCs in adult olfactory sensory neurons. Thus the reduced response to CO_2_ obtained from the antennae of TRPC RNAi strains is not due to a developmental defect.

**Conclusion:**

These observations show that reduction in TRPC channel function significantly reduces the sensitivity of the olfactory response to CO_2_ concentrations of 5% or less in adult *Drosophila*. It is possible that the CO_2_ receptors Gr63a and Gr21a activate the TRPC channels through Gαq and PLC21C.

## Introduction

Carbon dioxide (CO_2_), a green house gas, has context dependent effects on behavior of specific insect species. The moth *Manduca sexta* uses CO_2_ as a cue to evaluate flowers during foraging [1], [2] and ovipositioning [3]. Dipterans like the malaria mosquito, *Anopheles gambiae*, detect their host by following plumes of the host’s volatile emissions which contain CO_2_ [4]. The role of CO_2_ in determining *Drosophila* behavior in the wild is more complicated. CO_2_ was identified as one of the major components of the *Drosophila* stress odorant released by flies under stressful conditions (dSO) [5]. Other studies have shown that concentrations of CO_2_ as low as 0.1% act as a repellant for larval and adult *Drosophila* [6]. This repulsion can be masked by the presence of low concentrations of food and other odorants, a response presumably mediated by the need to reach fermenting food sources that also exude CO_2_ [6], [7]. The mechanisms by which *Drosophila* detects and responds to CO_2_ are therefore likely to be complex.

Low concentrations of CO_2_ (<10%) are sensed by two receptors, Gr21a and Gr63a, which co-express in the ab1C class of neurons housed in the large basiconic sensilla present on the third antennal segment of *Drosophila*. Flies lacking either of these receptors lose both electrophysiological and behavioral responses to CO_2_ [5], [8], [9]. The two *Drosophila* CO_2_ receptors, have corresponding homologues in mosquitoes referred to as GPRGR22 and GPRGR24, which co-express in the mosquito maxillary palps [9]–[11]. Thus, understanding the mechanism of sensory transduction downstream of CO_2_ receptors is of wide significance. The heterotrimeric G-protein Gαq has been implicated in the transduction of CO_2_ stimuli for concentrations of 5% or less [12]. The effectors downstream of Gαq in CO_2_ sensing neurons however remain elusive. One of the possible candidates could be the members of the canonical Transient Receptor Potential channel family (TRPC) which, from studies in *Drosophila* phototransduction have been known to act downstream of Gαq [13].

The TRP superfamily include a large number of cation channels [14] many of which are implicated in the detection and transduction of sensory information across a range of species (reviewed in [15]). In *Drosophila*, members of this superfamily have been implicated in the detection of a range of sensory stimuli including light [16]–[18], temperature [19]–[22], pain [23]–[25] mechanical stimuli [26], taste [27] and chemosensation [28]. Quite recently, a transient receptor potential channel was found to be involved in male-male courtship behavior in *Drosophila* [29]. In the *Drosophila* genome, the TRPC subfamily consists of TRP, TRPL and TRPγ, encoded by the genes *trp*, *trpl* and *trpγ* respectively. Of these the activity of TRP and TRPL are required to generate the light induced conductance in photoreceptors [30], [31]. In addition, hypomorphic alleles of *trp* (*trp^301^*) appear to have a defect in adaptation during responses to isoamylacetate and benzaldehyde [32]. In addition to TRP and TRPL, the *Drosophila* genome encodes a third member of the TRPC subfamily, namely TRPγ [33].

In *Drosophila* photoreceptors, the G-protein coupled receptor rhodopsin transduces photon absorption into the activation of TRP and TRPL channels. This transduction process requires the activity of the Gα subunit Gαq [13]. The activation of PLCβ (encoded by *norpA*) [34] by Gαq is an essential step in the activation of TRP and TRPL. While the subsequent steps in the mechanism of activation remain unresolved (reviewed in [35]), the requirement for G-protein coupled activation of PLCβ in TRP and TRPL channel activation can also be recapitulated in heterologous expression systems [36], [37]. Although the endogenous receptor and in vivo activation mechanisms of TRPγ remain unknown, when expressed in heterologous systems, TRPγ is reported to be activated downstream of receptors that trigger G-protein coupled PLC activity [33]. Thus the activation mechanism of *Drosophila* TRPC channels appears to have a conserved requirement for G-protein coupled PLCβ activity. In this study we investigated the possible role of genes encoding TRPC channels in *Drosophila* CO_2_ chemosensation.

**Figure 1:**
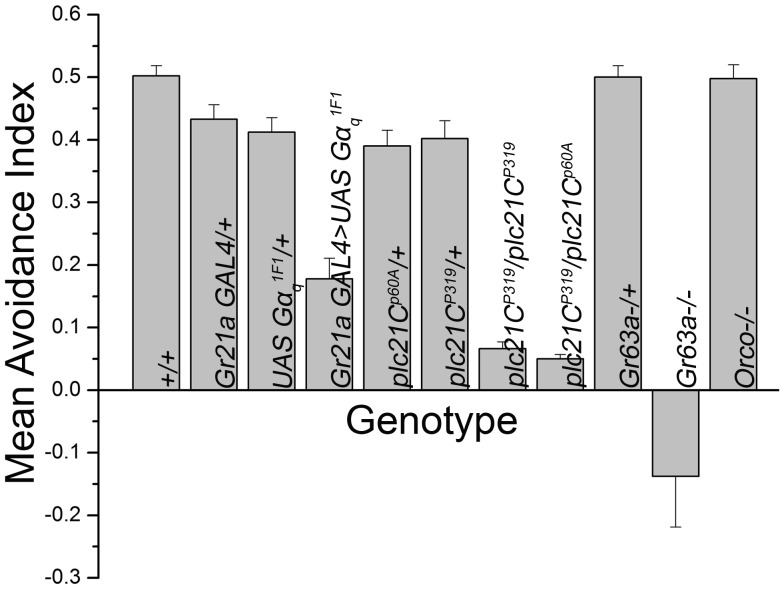
Disruption of *plc21C* gene leads to impaired CO_2_ sensing. Behavior analysis with 3 to 4 days old flies using the Y-maze with 5% CO_2_ and air shows reduced CO_2_ avoidance in flies homologous for the *plc21C* insertion allele *plc21C^P319^* (*plc21C^P319^/plc21C^P319^*) and heterologous with *plc21C* deficiency mutant *plc21C^p60A^* (*plc21C^P319^/plc21C^p60A^*). Heterozygous controls show normal behavioral avoidance (*p<*0.0001; two tailed student’s *t* test). *Gr63a−/−* flies were used as a negative control. Error bars indicate SEM.

## Materials and methods

### Fly Stocks

All flies were maintained at 25°C on standard corn meal agar medium unless specified otherwise. *Canton S* was used as the wild type strain. Other stocks used were *UAS Gq^1F1^ RNAi* [38], *plc21c^P319^* and *Df(2L)p60A* obtained from S. Leevers, UK [39], *Gr21aGAL4* on 3^rd^ chromosome received from Barry Dickson (Vienna, Austria), *Gr63aGAL4* on 2^nd^ chromosome, *GAL80^ts^*, *Gr63a^1^* (null allele of *Gr63a*), *Elav^C155^GAL4* on 1^st^ and *UAS RedStinger* on 3^rd^ from Bloomington stock centre, *UAS trpl RNAi* (VDRC 35571) and *UAS trpγ RNAi* (VDRC 9338) from Vienna Drosophila RNAi Center, *UAS H2bRFP* [40] from Boris Egger. *trp^343^/trp^343^*, *trpl^302^/trpl^302^* are published [41]. The *UAS trpl^+^)* strain was made by Amit Nair as follows. The *trpl* cDNA has been described earlier [31]. It was obtained as an EcoRI digested fragment from the parent plasmid and sub-cloned, into the *Drosophila* transformation vector *pUAST* [42]. Recombinant *pUAStrpl^+^* was used for generating stable transformants by standard procedures for microinjection of *Drosophila* embryos.

**Figure 2:**
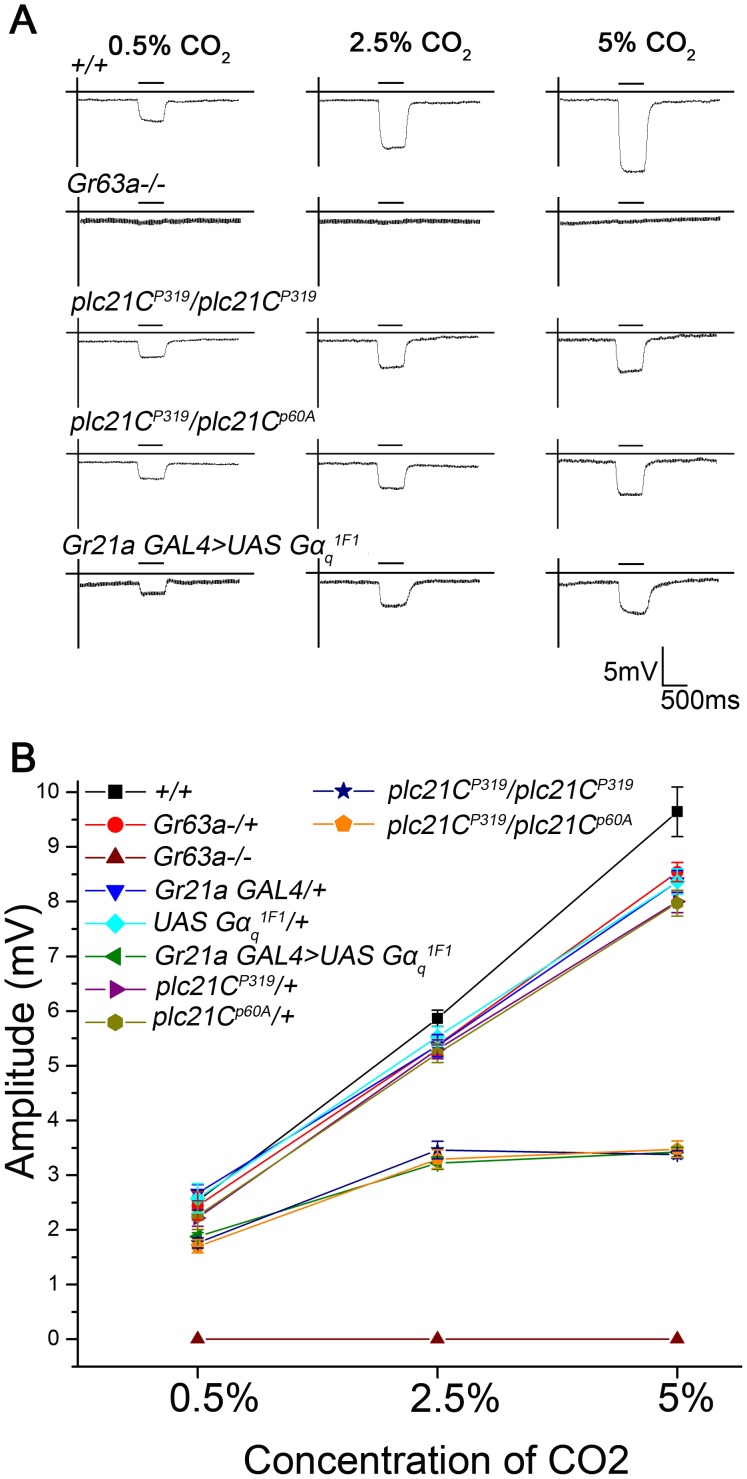
Electrophysiological recordings from the antennae of *plc21C* mutants. A) Representative traces of field recordings obtained from the basiconica rich region of the 3^rd^ antennal segment of 3 to 4 days old flies. Individual genotypes are indicated. Both the *plc21C* mutants show reduced electrophysiological responses to the three concentrations of CO_2_ tested as compared to the wild type flies (n = 10, *p<*0.0001). *Gr63a* null mutants (*Gr63a−/−*) and an RNAi knockdown of Gαq in CO_2_ sensitive neurons (*Gr21aGAL4>UASGαq^1F1^*) were included as test controls. B) Quantification of the field recordings for the genotypes tested (n = 10; *p<*0.0001). Error bars indicate SEM.

### Immunohistochemistry

*UAS H2bRFP* was driven in Gr21a receptor expressing cells in order to mark them. Frozen sections of the fly head (14 µm) were taken and stained with antibodies as previously described by Kain et al. [43]. The following primary antibodies were used; chick anti-RFP (1∶1000, Millipore), rat anti-TRP (1∶20). The antibody against *Drosophila* TRP was generated in house. The C-terminal 300 amino acids of TRP (aa 975–1275) were expressed as a His tagged fusion protein in *E. coli* and purified using Ni affinity chromatography. Purified antigen was used to immunize rats and generate a polyclonal antiserum. The specificity of the antiserum was tested using both Western blotting as well as immunohistochemistry using the *trp^343^* null allele as a control. Rabbit anti-TRPL (1∶100, catalog number AB5912 from Chemicon international) and rabbit anti-TRPγ (1∶300, obtained from Shireen A. Davies, University of Glasgow UK; [44]). Monoclonal antibody 22C10 (1∶5; DSHB) was used to mark the antennal sensory neurons. Secondary antibodies used were anti-chick, anti-mouse and anti-rabbit IgG conjugated to either Alexa 488 or Alexa 568 (1∶200; from Molecular Probes). Labeled samples were mounted in 70% glycerol or in an anti-fading agent, Vectashield (Vector labs) and examined in Olympus FV1000, at 1 µm slice intervals; data was processed using Image J, Confocal Assistant 4.2 and Adobe Photoshop 5.5. Whole antennal mounts were prepared using Vectashield (Vector labs) after fixing the antennae in 0.4% paraformaldehyde for 10 min followed by two washes in Phosphate buffered saline (PBS) of 10 minutes each. The samples were examined as stated above and the data was processed using FV10-ASW 3.0 viewer and Fiji (Image JA 1.45b) and Adobe Photoshop CS3 Extended.

**Figure 3:**
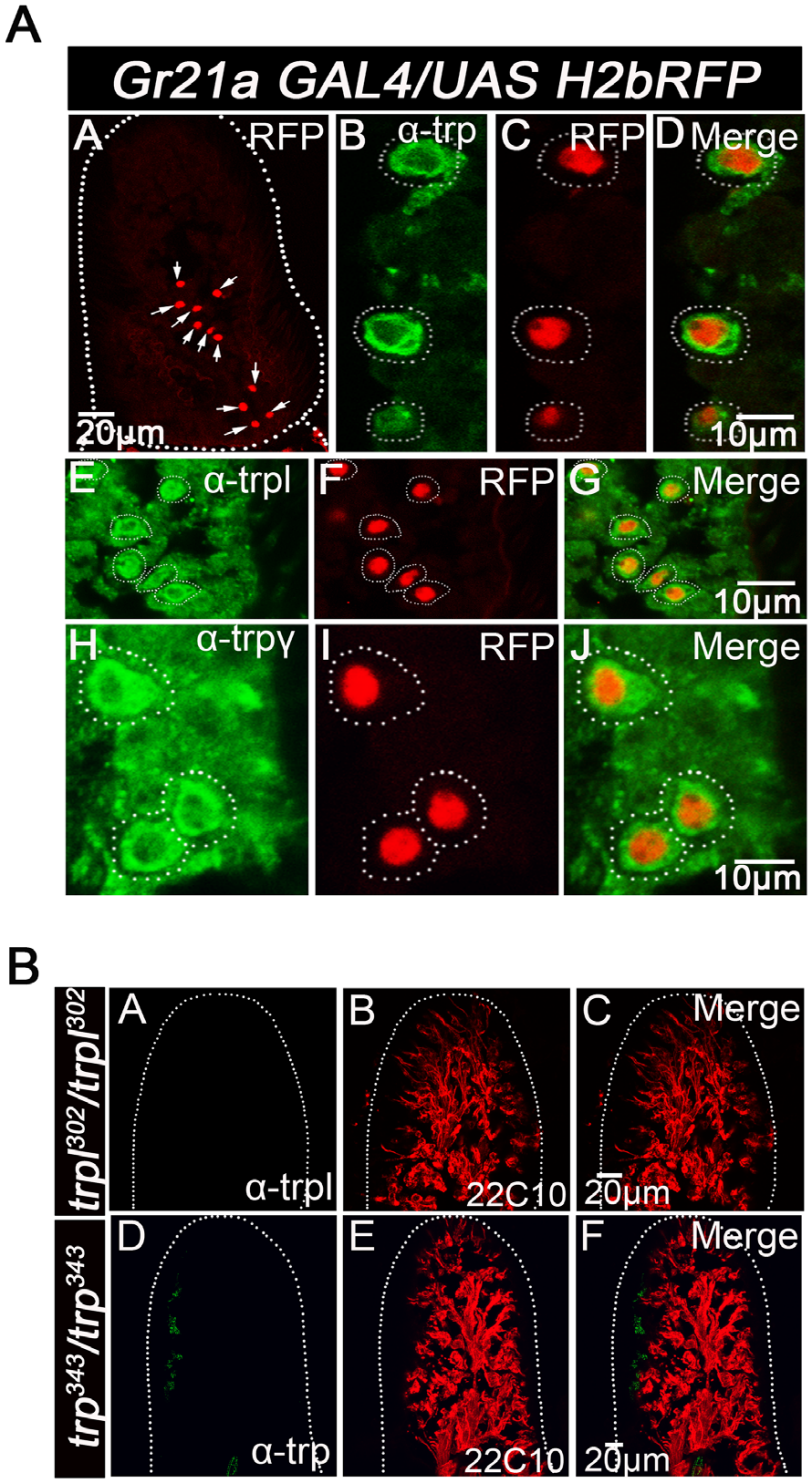
Expression of TRPC proteins in CO_2_ sensing neurons located in the third antennal segment of adult *Drosophila*. TRP, TRPL and TRPγ are expressed in CO_2_ responsive neurons in the adult *Drosophila* antenna. A) Frozen antennal sections (14 µm thick) from *Gr21aGAL4/UASH2bRFP* animals stained with anti-TRP, anti-TRPL and anti-TRPγ antibodies showing expression of TRP, TRPL and TRPγ respectively along the membranes of the Gr21a receptor neurons, marked by anti- RFP staining in red. The first panel shows the localization of Gr21a neurons in the antenna after staining with anti- RFP. B) Frozen antennal sections (14 µm thick) from the null mutants of *trpl* and *trp* stained with anti-TRPL and anti-TRP antibodies respectively. No expression of TRPL and TRP proteins could be observed in the respective mutant strains. mAb22C10 (anti-futch, microtubule protein) staining in red served as a neuronal marker.

**Figure 4:**
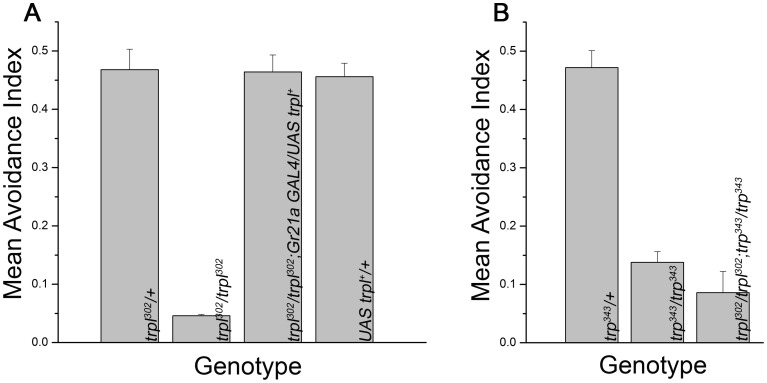
Null mutants of *trp* and *trpl* show reduced behavioral avoidance towards CO_2_. A) The mean avoidance index towards 5% CO_2_ in a Y-maze behavioral assay is shown for the indicated genotypes. The ability of *trpl* null homozygotes (*trpl^302^/trpl^302^*), to discriminate between 5% CO_2_ and air is significantly reduced (*p<*0.0001) as compared to the heterozygous control. The phenotype of the null mutant is rescued by expressing a wild type *trpl* transgene in Gr21a receptor neurons (*trpl^302^/trpl^302^; Gr21a GAL4/UAS trpl^+^*) (*p<*0.0001). B) Null mutant of *trp* (*trp^343^/trp^343^*) has reduced avoidance to 5% CO_2_ in the Y-maze assay. The avoidance response of the double null mutant (*trpl^302^/trpl^302^; trp^343^/trp^343^*) is also reduced but not significantly different from the single null homozygotes (*p>*0.05). Error bars indicate SEM in A and B.

### Electrophysiology

Extracellular field recordings were acquired from the large basiconica rich region on the third antennal segment of the fly antenna [8] using DIGIDATA 1322A 16-Bit Data Acquisition System (Axon Instruments) connected to a DAM 50 Differential amplifier (World Precision Instruments) using borosilicate glass electrodes of 30–35 MΩ resistance (GC100F-10; Harvard Apparatus Ltd.) containing 0.8% NaCl and a 0.250 mm silver wire (AGW1010; World Precision Instruments). The stimulus was delivered as a 500 ms pulse at a flow rate of 1L per minute. Three different concentrations of CO_2_, 0.5%, 2.5% and 5% were achieved by diluting 100% CO_2_ in air and the concentrations were confirmed using a CO_2_ sensor (Type-IR-CO_2_ gas tester; Heraeus). Air was used as a negative control in addition to being flushed along the delivery tube between each concentration shift to minimize CO_2_ accumulation. Flies were allowed to rest for one minute between concentration shifts to avoid adaptation effects. Electroretinograms were recorded from the eyes of flies using 5 s pulses of green light. All traces were analyzed using Clampfit Version 9.0.1.07 software (Axon Instruments). All flies used for electrophysiology were 3 to 4 days old females. A minimum of 10 flies per genotype were tested.

**Figure 5:**
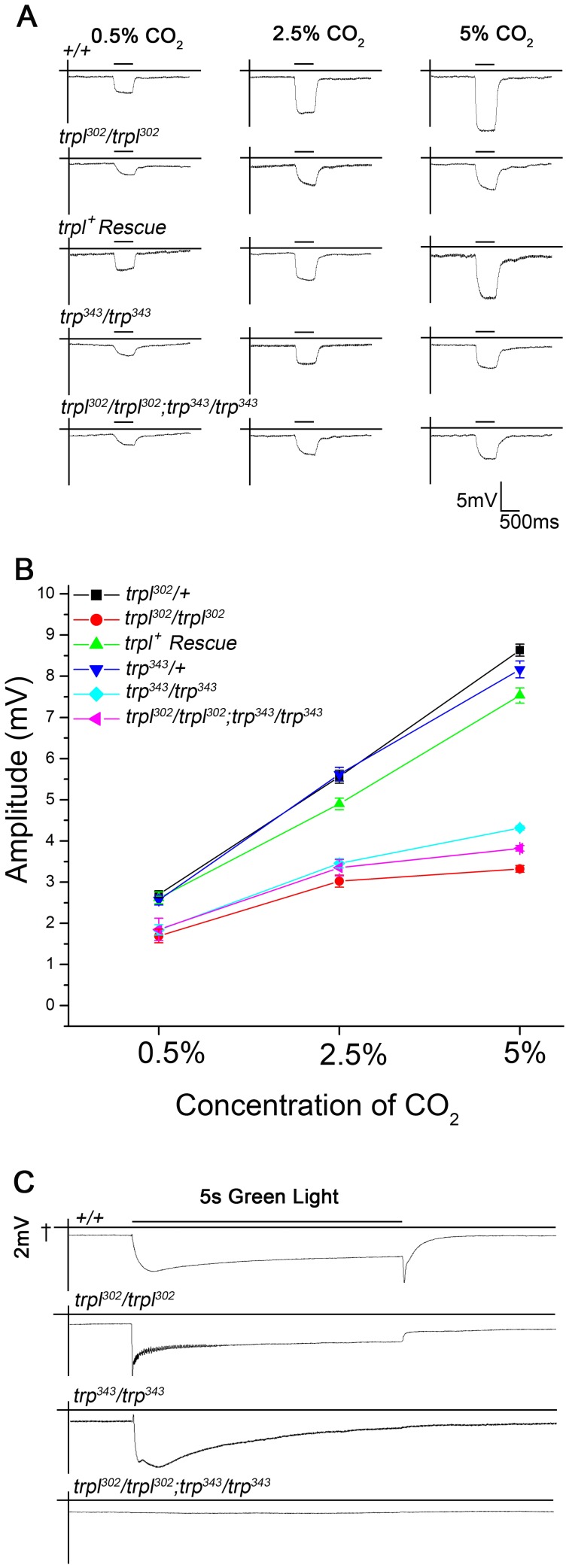
Electrophysiological responses to various concentrations of CO_2_ obtained from antennae of wild type and mutants. The response from *trpl* null homozygotes (*trpl^302^/trpl^302^*), *trp* null homozygotes (*trp^343^/trp^343^*) and the double null mutants (*trpl^302^/trpl^302^;trp^343^/trp^343^)* appear reduced towards all three CO_2_ concentrations tested as compared to wild type (*+/+*) responses. A) Representative traces of field recordings obtained as described above. B) Quantification of the field recordings for various mutant and control genotypes tested. Both *trpl* null (*trpl^302^/trpl^302^*) and *trp* null mutants (*trp^343^/trp^343^*) along with their double mutant (*trpl^302^/trpl^302^; trp^343^/trp^343^*) show reduced electroantennogram responses (n = 10; *p<*0.0001). The reduced response of the *trpl* null is rescued by the expression of wild type TRPL in Gr21a receptor neurons (n = 10; *p<*0.0001). *Gr63a−/−* served as the negative control with no EAG response. C) Photoresponses of the indicated *trpl* and *trp* mutants. n = 10 for all genotypes shown. Error bars indicate SEM.

### Behavioral Analysis

The Y- maze set up, as described by Das et al. [45], was used to carry out behavioral assays and the Mean avoidance index was calculated as described [9] as the number of flies in the CO_2_ arm subtracted from the number of flies in the air arm divided by the total number of flies in both arms. Flies that did not choose either arm were not taken into consideration. The concentration of CO_2_ used was 5%. Each experimental set contained 25 to 30 flies of 3 to 4 days of age and ten experimental sets were used per genotype. All genotypes tested were double blinded. The orientation of the arms of the Y-maze was alternated to avoid any side bias.

**Figure 6:**
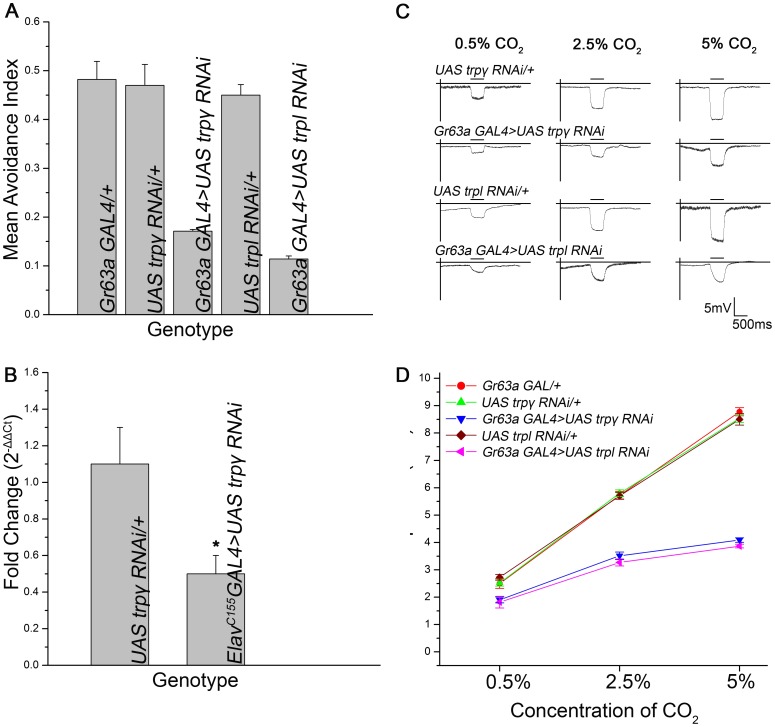
Down regulation of *trpl* and *trpγ* in CO_2_ receptor neurons results in reduced sensitivity to CO_2_ as observed in the responses from the Y-maze behavioral assay with 5% CO_2_ in A (*p<*0.0001). B) qRT-PCR data showing the fold change of *trpγ* gene expression in the *UAS trpγ RNAi* line relative to its control as determined by the comparative ΔΔCt method (N = 6; *p<*0.05). C) Representative traces of field recordings obtained as described above. Individual genotypes are indicated. D) Quantification of the field recordings for the genotypes tested (n = 10; *p<*0.0001). Error bars indicate SEM.

### Data Analysis

Two tailed student’s *t* test was used to compare heterozygous controls with their corresponding homozygous knockout and knock down lines in all molecular, electrophysiological and behavioral experiments.

**Figure 7:**
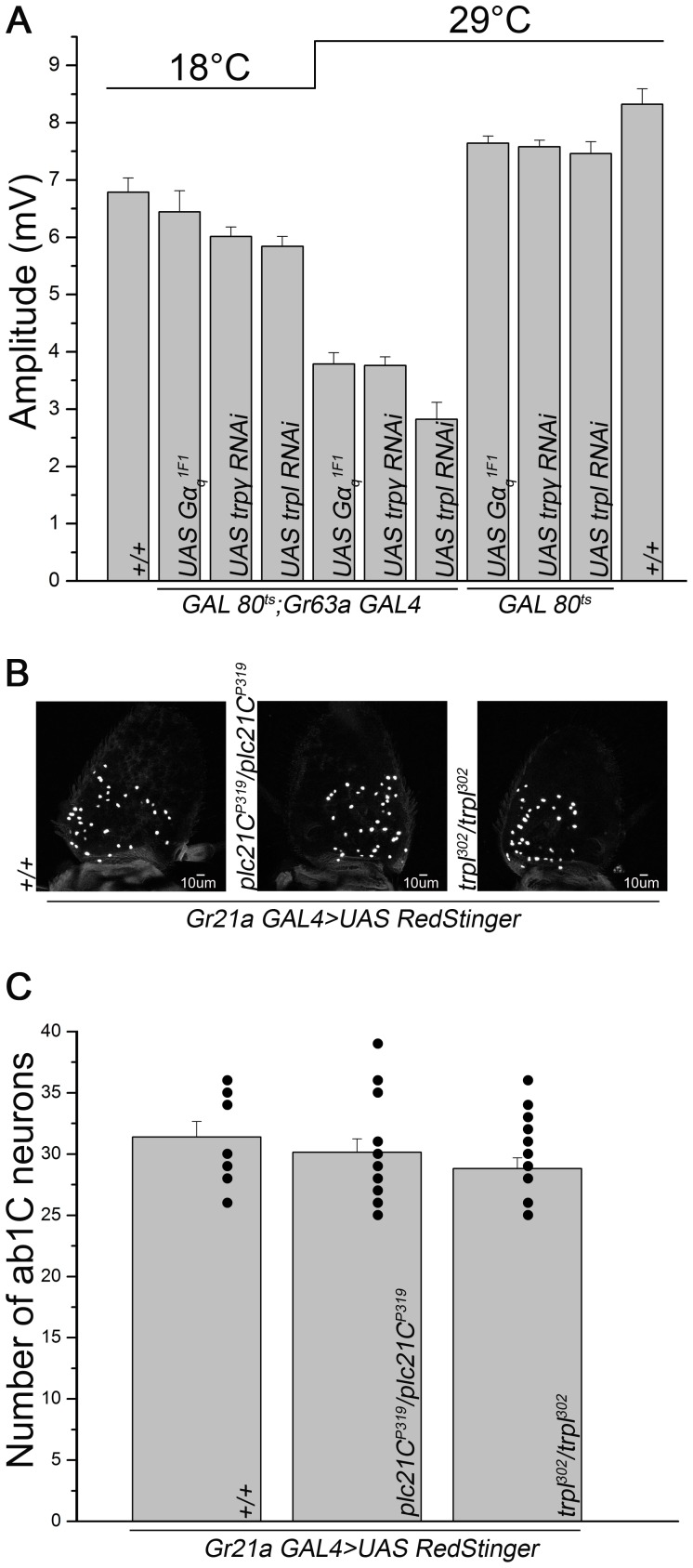
Reduced sensitivity to CO_2_ is not a developmental defect. A) RNAi lines grown at the restrictive temperature of 18°C (active GAL80) show normal electrophysiological responses to CO_2,_ since the CO_2_ receptor neuron specific GAL4 remains inactive (absence of RNAi expression; *p>*0.05). RNAi lines grown at the permissive temperature of 29°C (inactive GAL80) show reduced electrophysiological responses to CO_2_ due to active GAL4 and RNAi expression (n = 10; *p<*0.0001). The RNAi heterozygotes in the absence of *Gr63aGAL4* show normal responses to CO_2_ at 29°C. Error bars indicate SEM. B) Whole antennal mounts showing CO_2_ sensory neurons marked using *UAS RedStinger* driven by *Gr21aGAL4* in wild type, *plc21C^P319^/plc21C^P319^* and *trpl^302^/trpl^302^* mutant lines. C) Quantification of CO_2_ sensory neurons in the adult antennae of the same lines (n = 14; *p* value not statistically significant).

### Relative Quantitation of Gene Expression

250 µg of RNA was extracted from 10 *Drosophila* 3^rd^ instar larval brains per sample set with 6 sample sets in total per genotype. Reverse transcription PCR (RT-PCR) was performed as described in [46]. Real-time quantitative PCR (qPCR) was performed on 1∶10 dilution of the total cDNA with duplicates per sample set using *rp49* primers as internal control and primers specific to the gene of interest (*trpγ*) on the 7500 Fast Real-Time PCR System (Applied Biosystems) operated with 7500 software v2.0.5 using MESA GREEN qPCR™ Master Mix Plus for SYBR® Assay - dTTP (Eurogentec, Belgium).

### Experiments with Temperature Sensitive *GAL80*

Flies of the appropriate genotypes were maintained at the restrictive temperature of 18°C until eclosion and then transferred to the permissive temperature of 29°C when the RNAi was allowed to express. These flies were then used to carry out electrophysiological recordings after ageing for 3 to 4 days.

## Results

### Phospholipase Cβ Encoded by *plc21C* is Required for Normal Avoidance Behavior of CO_2_

The *Drosophila* genome contains two genes encoding phospholipase Cβ referred to as *norpA* [34] and *plc21C* [47]. Previous studies have shown that *norpA* is not required for either behavioral or physiological responses to CO_2_ [12]. When two mutants of *plc21C, plc21C^P319^* (an insertion allele) and *plc21C^p60A^* (a deficiency line), were tested for their response to 5% CO_2_ in a Y- maze behavioral assay both showed reduced avoidance (Fig. 1, *p<*0.0001). *Canton S (CS)* flies were used as positive controls and these showed normal avoidance towards 5% CO_2,_ while null alleles for the CO_2_ receptor, *Gr63a,* showed complete impairment in CO_2_ sensing as demonstrated previously [9] (Fig. 1). As expected, knock down of Gαq in CO_2_ sensory neurons (*Gr21aGAL4>UASGαq^1F1^*) by a previously tested RNAi construct also reduced the avoidance response of adult *Drosophila* towards CO_2_ [12], [38]. The specificity of the behavioral response was further verified by testing the response of *Orco* null mutants to 5% CO_2_. These flies showed normal levels of avoidance towards CO_2_ (as previously observed by Turner et al. [7] Fig. 1). The *Orco* gene product is a highly conserved atypical member of the olfactory receptor family and serves as a co-receptor for olfactory receptors in *Drosophila* [48]. It is expressed in a majority of olfactory sensory neurons of the antenna but not in CO_2_ sensing neurons [8], [48].

Electrophysiological responses were obtained from the region of the third antennal segment housing the large basiconic sensilla containing the ab1C neurons [8]. Both *plc21C* mutant alleles tested, *plc21C^P319^/plc21C^P319^* and *plc21C^P319^/plc21C^p60A^* showed lowered sensitivity to all the three CO_2_ concentrations, thus corroborating the results observed during behavior analysis (Fig. 2A and B, *p<*0.0001).

### TRPC Proteins are Expressed in CO_2_ Receptor Neurons of the Adult *Drosophila* Antenna

From previous studies in *Drosophila* photoreceptors, it is known that TRPC channels TRP and TRPL are activated by Gαq stimulation of PLCβ [13], [34]. Therefore, the presence of TRPCs was ascertained in the third segment of adult *Drosophila* antennae, which host a majority of olfactory sensory neurons including those for CO_2_. The expression of each of these channels was determined in adult *Drosophila* antennae by immuno-staining with antibodies specific for each TRPC protein. As shown in Fig. 3A, *Drosophila* TRP, TRPL and TRPγ were indeed expressed in the third antennal segment of the fly. Their presence in CO_2_ sensory neurons was confirmed by marking the nuclei of these with a Histone2b Red Fluorescent Protein (H2bRFP) fusion construct [40]. Cellular localization of TRPCs appeared to be on cell membranes of neurons with H2bRFP expressing nuclei. Thus the CO_2_ receptor neurons of adult *Drosophila* express TRP, TRPL and TRPγ. Null mutants of *trp* and *trpl* were used as negative controls to validate the specificity of the antibodies (Fig. 3B).

### Null Mutants of *trp* and *trpl* Show a Reduced Behavioral Response Towards CO_2_

To understand the functional role of TRPCs in olfactory responses to CO_2,_ protein null mutants in the *trp (trp^343^)* and *trpl (trpl^302^)* genes were studied. Homozygous *trpl^302^* when tested for their avoidance to 5% CO_2_ in a Y-maze gave a mean avoidance index of just 0.04, as compared to 0.46 obtained for *trpl^302^* heterozygotes (Fig. 4A, *p<*0.0001). In order to confirm that the reduced response to CO_2_ in *trpl^302^* flies is indeed due to the mutation in the *trpl* locus, a wild type *trpl* transgene [*UAS trpl^+^*] was expressed in the CO_2_ receptor neurons of *trpl^302^/trpl^302^* null flies. The behavioral avoidance towards CO_2_ was restored back to 0.45 in *trpl^302^/trpl^302^;Gr21aGAL4/UAS trpl^+^* animals (Fig. 4A). Interestingly, the behavior of null mutants of *trp* (*trp^343^/trp^343^*) towards 5% CO_2_ was also found to be reduced (0.13) although slightly higher than that observed for the *trpl* null mutants (Fig. 4B, *p<*0.0001). The behavioral phenotype of *trpl^302^/trpl^302^;trp^343^/trp^343^* double mutants was also measured. This was not significantly different from the individual null mutants (Fig. 4B, *p>*0.05).

### Electrophysiological Recordings from *trpl* and *trp* Null Antennae Correlate with their Mutant Behavior Towards CO_2_

In *trp* and *trpl* null mutants, the altered behavior towards CO_2_ could arise from either a reduction in CO_2_ sensing by peripheral sensory neurons or by changes in central brain circuits responsible for the CO_2_ avoidance behavior. While rescue by expression of *UAS trpl^+^* in the CO_2_ sensory neurons suggested that the primary defect was in the periphery, this was further tested by measuring electrophysiological responses, to varying concentration of CO_2,_ from the antenna. A consistent reduction in the amplitude of electro-antennogram responses of *trp^343^/trp^343^* and *trpl^302^/trpl^302^* flies was observed in comparison to wild type and heterozygous controls. Reduced responses were observed for all three concentrations of CO_2_ (Fig. 5A and B, *p<*0.0001). Expression of the *UAS trpl^+^* transgene with *Gr21aGAL4* in the *trpl^302^/trpl^302^* flies rescued the electrophysiological phenotype significantly, further confirming a role for *trpl* in CO_2_ sensory neurons (Fig. 5A and B). Consistent with the behavioral results, the electrophysiological responses for *trpl^302^/trpl^302^;trp^343^/trp^343^* double mutant flies were similar to that of the individual null alleles (Fig. 5A and B). These data suggest that the lowered sensitivity to CO_2_ is indeed due to a reduction in CO_2_ sensing by the peripheral sensory neurons and not by changes in central brain circuits. It is also evident from the data presented that TRP and TRPL are not the only channels that function in response to CO_2_ in Gr63a and Gr21a positive sensory neurons.

To confirm the genotypes of the TRPC mutants, electroretinogram responses (ERGs) were measured as described in materials and methods. For each genotype, the responses obtained were similar to the published data where it has been shown that a null mutant of *trp* shows only a transient response to prolonged light stimulus and a null mutant of *trpl* has oscillations superimposed on its response (Fig. 5C) [49]. Importantly, there was no response seen in *trpl^302^/trpl^302^;trp^343^/trp^343^* to the light stimulus [31]. In contrast, the residual responses to CO_2_ observed in *trpl^302^/trpl^302^;trp^343^/trp^343^* animals suggest that the physiological role of the two TRPC channels, TRP and TRPL, in CO_2_ sensing neurons is different from what has been observed in the photoreceptors [31], [49].

### Down-regulation of *trpγ* and *trpl* in CO_2_ Receptor Neurons Leads to Impaired CO_2_ Sensing

Next the effect of down-regulating TRPγ, the third TRPC channel in *Drosophila* was assessed on CO_2_ driven behavior and electrophysiology. For this purpose we used the *Gr63a GAL4* strain to drive expression of UAS driven RNAi lines for *trpγ* and *trpl,* so as to knock down these genes specifically in CO_2_ sensory neurons. Flies with down-regulation of either *trpγ* or *trpl* in Gr63a expressing neurons were relatively indifferent to 5% CO_2_ (Fig. 6A). In both cases the responses were significantly different from the controls (*p<*0.0001). The mean avoidance index of *trpl* knockdown flies was 0.1 while *trpγ* was 0.15. These values are comparable to the avoidance index of *trpl* null mutants in figure 1. In all cases the avoidance index of controls was equal to or greater than 0.45. In order to validate the RNAi line for *trpγ*, qRT-PCR was carried out on RNA extracted from third instar larval brain samples of the *UAS trpγ RNAi* line driven by a pan neuronal GAL4 (*Elav^C155^GAL4*) as described in the materials and methods. The RNAi line for *trpγ* showed ∼45% reduction for the *trpγ* cDNA when compared to its control (Fig 6B, *p<*0.05). Direct validation of the efficacy of the RNAi line was not possible in the CO_2_ receptor neurons due to their low count (25–35 neurons) within the antennae.

Electrophysiological field recordings from *trpγ* and *trpl* knockdown strains confirmed their inability to sense CO_2_ at the same sensitivity as wild-type or control flies. (Fig. 6C, D). At all three concentrations of CO_2_, electrophysiological responses were significantly reduced (*p<*0.0001). Thus the ability of adult *Drosophila* to sense and respond to CO_2_ in the environment depends to a significant extent on the three TRPC channels, TRP, TRPL and TRPγ.

### Reduced Responses to CO_2_ is not a Developmental Defect

To test if the reduced sensitivity to CO_2_ is a consequence of TRPC channel function in adult sensory neurons or due to unidentified developmental changes in the olfactory circuit for CO_2_, expression of the UAS RNAi lines for *trpl* and *trpγ* was limited to adult sensory neurons with the help of a temperature sensitive *GAL80* transgene which renders *GAL4* inactive at 18°C. At the non-permissive temperature of 29°C the *GAL80* can be inactivated thus enabling *GAL4* to drive the RNAi [50]. Electrophysiological responses of flies in which *UAS trpl RNAi* and *UAS trpγ RNAi* were expressed after eclosion showed reduced responses to CO_2_ as compared to controls and flies grown exclusively at 18°C (Fig. 7A, *p<*0.0001). These data confirm that reduced CO_2_ responses in flies with RNAi knockdown of *trpl* and *trpγ* occurs due to the reduction of the individual TRPC proteins in adult antennal sensory neurons and is not due to developmental changes.

Furthermore, adult antennal CO_2_ sensory neurons were quantified in different mutant backgrounds (*plc21C^P319^/plc21C^P319^* and *trpl^302^/trpl^302^*) by driving *UAS RedStinger* in Gr21a receptor neurons to mark their nuclei. The CO_2_ sensory neuron counts were found to be within the normal range (approximately 25 to 30 neurons) [5], [8] and similar to the wild type control (Fig. 7B and C, *p* value not statistically significant). These observations show that reduced CO_2_ sensing by the various mutant lines is not due to a reduction in CO_2_ sensing neurons during development. These observations further implicate TRPC channels as components that determine the high sensitivity of adult *Drosophila* CO_2_ sensory perception.

## Discussion

The role for TRPC channels in maintaining the high sensitivity of CO_2_ detection is important in multiple contexts. Detection of low concentrations of CO_2_ (5% or less) shares several similarities with odor detection. Receptors for low concentrations of CO_2,_ despite belonging to the gustatory class of insect chemosensory receptors, are located within olfactory sensillae on the third antennal segment. Moreover, mutants in *dgq*, the gene that encodes the α subunit of the heterotrimeric G-protein Gαq, reduce the physiological response recorded from sensory neurons in both cases [12], [43]. We now show that mutants of the ubiquitously expressed allele of PLCβ, *plc21C* [47] reduce the response to CO_2_ similar to the observation for odors [43] unlike mutants of *norpA* allele which is expressed strongly in the eyes and is required for phototransduction [34] but not for CO_2_ sensing [12]. In olfactory sensory neurons it has been proposed that the physiological response to odorants is a combination of ionotropic and metabotropic receptor signaling. The olfactory receptor and olfactory co-receptor (Or/Orco) complex forms an odor-activated ion channel [51], [52] in heterologous systems and is therefore thought to be an ionotropic component, while the olfactory receptor coupling to a G-protein, like Gαq, could initiate the metabotropic component through as yet un-determined ion channels. Unlike olfactory sensory neurons, ab1C, the CO_2_ sensing neurons do not express the olfactory receptor and olfactory co-receptor (Or/Orco) complex. Therefore, in these neurons it is possible that the ionotropic component is absent. Our data suggest that TRPCs, which are known to function downstream of Gq/Plcβ signaling [13], [33], [34], [36], [37] may contribute to metabotropic signaling in ab1C neurons but our data does not allow us to state this conclusively. However it is evident that the TRPC channels are required for the normal functioning of CO_2_ sensing ab1C neurons in adult *Drosophila*. The presence of a basal response in individual knock outs and knock downs of *trp*, *trpl* and *trpγ* and double null mutants of *trp* and *trpl* as compared to the complete lack of response in *Gr63a* null flies suggests that the CO_2_ sensing ab1C neurons are not solely dependent on the TRPC channels for function. While it is formally possible that the remaining response in *trpl^302^;trp^343^* double nulls is due to *trpγ*, we do not favor this idea primarily because, the response of double mutant nulls was no worse than that of single mutants. The triple mutant combination of *trpl^302^;trp^343^* with the *trpγ* RNAi line was poorly viable and hence could not be tested directly.

The consequences of this finding are relevant for *Drosophila* behavior. Unlike other insect species like moths and mosquitoes, *Drosophila* are innately repelled by low concentrations of CO_2_ presumably because it is an indicator of stress due to a potential threat to naïve flies. However, in conditions where CO_2_ is present along with food odorants this repulsion needs to be suppressed. Our data suggest that TRPC channels are a component of this dual sensitivity. Repression of Gq/PLCβ signaling and/or TRPCs through mechanisms yet to be identified might reduce the sensitivity to CO_2_ and alter the behavior from repulsion to attraction. Interestingly, food odors that can reduce CO_2_ responses from ab1C neurons have been identified [7]. Whether these odorants act through repression of TRPCs needs to be determined. Thus it appears that the three *Drosophila* TRPC channels TRP, TRPL and TRPγ can act as amplifiers of the signal downstream of a channel yet to be identified while playing redundant roles in this amplification process. The requirement for redundancy might stem from an evolutionarily conserved need to escape stress and or the necessity to find food.
